# Enhancing clinical practice through action research: fostering a person-centred culture in healthcare

**DOI:** 10.3389/frhs.2025.1583478

**Published:** 2025-06-06

**Authors:** Elizabeth Rosted, Helle Gert Christensen, Tina Lanther, Brendan McCormack, Mette Kjerholt

**Affiliations:** ^1^ Faculty of Health Sciences, University of Southern Denmark, Odense, Denmark; ^2^ Department of Oncology and Palliative Care, Zealand University Hospital, Roskilde, Denmark; ^3^ Department of Hematology, Zealand University Hospital, Roskilde, Denmark; ^4^ Susan Wakil School of Nursing and Midwifery, Faculty of Medicine and Health, The University of Sydney, Sydney, NSW, Australia

**Keywords:** person-centred care, person-centred culture, action reseach, participatory, implementation

## Abstract

**Introduction:**

Despite the growing recognition of person-centred practice in political declarations, strategic visions, and healthcare policies, a significant gap remains between the theoretical underpinnings of person-centred practice and its practical application in clinical settings. To bridge this gap, two departments at a university hospital in Denmark embarked on a collaborative initiative aimed at developing a person-centred culture and implementing a person-centred approach as the framework for nursing care.

**Method:**

An action research study was undertaken from June 2020 to December 2023. To capture both the processes and outcomes of the study, data were collected through a combination of field studies, interviews and questionnaires. Data were analysed using thematic analysis.

**Results:**

Five themes were generated from the data (1) We have learned a lot in relation to PCP; (2) The PCP framework is complex and difficult to understand and get hold of; (3) When implementing a person-centred framework, the overall context of the departments is important; (4) Psychological safety is important; (5) Values and Beliefs.

**Conclusion:**

Overall, the study findings showed outcomes arising from the development of a caring culture towards one that is more person-centred. Both nurses and leaders’ competencies in relation to PCP were developed. The study resulted in increased awareness, knowledge and learning of PCP and during the study period new initiatives were initiated that contributed to the changes in and of clinical practice towards a more person-centred culture. During the study, blind spots like a disconnect between espoused values and experiences in clinical practice, as well as inappropriate workflows in the departments and the organization were uncovered.

## 1 Introduction

In recent years, the concept of person-centred practice (PCP) has emerged as a cornerstone of modern healthcare systems. This, particularly in countries like Denmark, where the health-care framework is increasingly oriented towards humanising medical interactions and practices.

The Danish healthcare system, with its foundational ethos of person-centredness, strives to respect and address the individual needs of every patient. This commitment is encapsulated in the vision and strategy of local hospitals, which underscore the significance of tailoring treatment and care to the unique context of each patient. The strategic framework for nursing at the University Hospital, where this study is hosted, articulates a clear vision for 2025 emphasising that care and treatment must be person-centred and the necessity of adapting care to the local context emphasised (1). This strategic focus is consistent with the patients' expectations for their involvement in decisions regarding treatment and care (2).

In oncology, PCP is increasingly recognized as an essential approach to care, as it emphasizes the individual needs, preferences, and values of patients (3). Research by Barker et al. (4) and Nkhoma et al. (5) highlights that a person-centred approach enhances patient wellbeing (4, 5) and satisfaction with care (6–9). Studies have consistently demonstrated that when patients are actively engaged in their treatment decisions, they experience a greater understanding of their disease and treatment options, which in turn fosters adherence to prescribed treatment plans (7, 10). This alignment between patient involvement and treatment adherence is vital, as it can lead to more judicious use of healthcare resources, ultimately yielding positive economic outcomes for healthcare systems (5, 10–12). Moreover, the benefits of PCP extend beyond individual patients to encompass the families of those affected by cancer. Involving patients in their care not only alleviates the burden on family members but also enhances their satisfaction with the care process (10). This interconnectedness between patient involvement and family satisfaction underscores the holistic nature of PCP, which prioritises the well-being of the entire support network surrounding the patient.

The emphasis on humanity, respect, mutual communication and holistic interactions within clinical care inherent in PCP is not only beneficial for patients but also significantly impacts healthcare professionals. A focus on PCP values can significantly enhance nurses' work satisfaction as value-aligned work protects nurses from experiencing burnout (13, 14). Conversely, when healthcare workers are compelled to set aside their personal values in the workplace, it poses a significant risk for their well-being and retention (15, 16).

Despite the growing recognition of PCP in political declarations, strategic visions, and healthcare policies, there remains a significant gap between the theoretical underpinnings of PCP and its practical application in clinical settings. While PCP is frequently discussed as an ideal approach, its translation into practice often falls short (17), or it is limited to reported accounts of shared decision making (17).

To bridge this gap, an oncological department and a haematological department at a university hospital in Denmark have embarked on a collaborative initiative aimed at developing and implementing a person-centred approach as the framework for nursing care. This was based on two researchers theoretical and practical knowledge of PCP and their experiences in clinical practice. Experiences were that clinical practice lacked a common theoretical framework and shared values, which resulted in confusion and sometimes even disagreement when reflecting on clinical practice. Consequently, care was guided by the nurses' individual, implicit values and beliefs. The researchers recommended that the chief nurses would support a study to develop a person-centred culture in accordance with the understanding of McCance and McCormack (18). The chief nurses supported and consented to the study, resulting in a top-down decision to develop and implement a person-centred culture. Despite the top-down nature of the decision, co-researchers found the study meaningful and were eager to participate in order to enhance their practice from a bottom-up perspective. The PCP framework was used as a “heuristic device” to help expand clinicians' mindsets and support them to see the meaning and significance of entering healthful relationships with all care providers and service users, underpinned by values of respect for persons, individual rights to self-determination, mutual respect and understanding (19). All five domains of the PCP framework were presented to all clinicians, but the particular focus in the project described here was on prerequisites, the person-centred processes and the practice environment.

Thus, this initiative was not merely an exercise in compliance with policy but rather a concerted effort to embed the principles of PCP into cancer care. By adopting the Person-centred Practice Framework of McCance and McCormack (18) the departments' aim to prioritise nursing care in accordance with PCP while simultaneously fostering a supportive environment for healthcare professionals had the potential to be realised.

The purpose of this paper is to detail the development and implementation of a person-centred practice framework in the context of cancer care, highlighting the processes undertaken, the challenges encountered, and the outcomes achieved. Through this exploration, we aim to contribute to the growing body of literature on person-centred care in oncology, providing insights that may inform future practices and policies in the healthcare sector.

## 2 Aim

The aim of this study was:

To develop a person-centred culture in the two departments, whereby all nurses would gain the competence to provide nursing care in accordance with PCP principles and practice respectful relationships with colleagues, resulting in a healthful culture.

To achieve this aim, we identified four research questions:

1. How do clinical nurse specialists and nurse leaders experience the process of implementing PCP?
2. How does the action research design support the development of co-researchers' competence?
3. How do clinical nurse specialists and nurse leaders experience PCP and the culture after the implementation process?
4. How do nurses experience nursing care in the departments?
5. How do patients experience nursing care in the departments?

## 3 Method

A participatory action research study was undertaken from June 2020 to February 2024, as participatory action research is appropriate for developing and transforming cultures as well as developing participants' competence (20). The research involved participants throughout the research process, from initial planning to implementation and evaluation and it was characterised by a continuous cyclical framework, delineated into the four phases of identification, planning, implementation, and evaluation (21, 22), illustrated in Table 2. These phases were closely interwoven, with cyclical activities of planning, taking action, observing and reflecting and evaluation occurring within each phase (20). AR can greatly develop the participants' competencies, as it combines practical action with reflection and action in a cyclical process (20). The study was conducted in accordance with a person-centred approach considering connectivity, drawing on attentiveness, dialogue, empowerment and participation as well as critical reflexivity (23), illustrated in Table 1.

**Table 1:** The study was conducted in accordance with a person-centred approach to research considering connectivity, drawing on attentiveness, dialogue, empowerment and participation as well as critical reflexivity (23).

Principles of person-centred research	Description and actions of the researchers
Connectivity	Connectivity refers to the view that it is out of relationships that we as human beings grow and flourish and that knowledge, too, is co-constructed in the coaction of people; not linear and causal influence, but confluence as a relational view of agency is key in connectivity. *The researchers were aware of opening up to new possibilities, perspectives and ways of being during our dialogues on person-centredness and culture in clinical practice.*
Attentiveness and dialogue	Attentiveness is a matter of seeing oneself, others, contexts and their interrelationship. *It requires from the researchers, the capacity to be contextually aware, listen, and to see and to interpret systematically what we hear and what we see.* Dialog is based on mutual attention and respect: recognizing oneself and the other as a person that is worthwhile, which lends dignity and autonomy to the other and oneself. *Dialog requires openness and transparency in relation to what or whom we attend to*. *During dialogs and meetings, the two researchers attempted openness and transparency, and they spent time to reflect on oneself, others, contexts and their interrelationship.*
Empowerment and participation	Individuals and groups should be empowered by facilitating development into self-awareness and self-esteem, capacity building and action. *Participants were involved in all phases and processes throughout the study, from initial planning to implementation and evaluation and they gained insights, knowledge and concrete tools according to facilitators and barriers for developing a person-centred culture.*
Critical reflexivity	Critical reflexivity is needed to understand what power relationships are fostered and maintained and who benefit from them. Researchers should be aware of their own positions and interests and to explicitly situate themselves within the research. *As reflexive researchers, the researchers looked at the research process, the context and the outcome and during dialogue meetings with co-researchers we invited to dialogs on power-relationships and safe spaces.*

### 3.1 Setting

The setting was two departments treating patients with cancer at a University Hospital, in Denmark. One department was the Department of Oncology and Palliative care located at three different hospitals in the region, where 168.000 treatments and consultations are carried out every year. The vision for nursing practice was based on holistic and humanistic values defined by Dame Cicely Saunders (24) and Professor Kari Martinsen (25). The other department was the Department of Haematology located at one hospital performing 69.000 consultations every year. The department was established in 2011 and the vision for nursing practice was based on a holistic, humanistic and participatory approach (21). As the Department of Haematology had been working with a participatory approach, they were more familiar with involving both nurses, leaders and patients in development processes. Nevertheless, the two departments shared the study aim.

### 3.2 Participants

**Figure 1:**
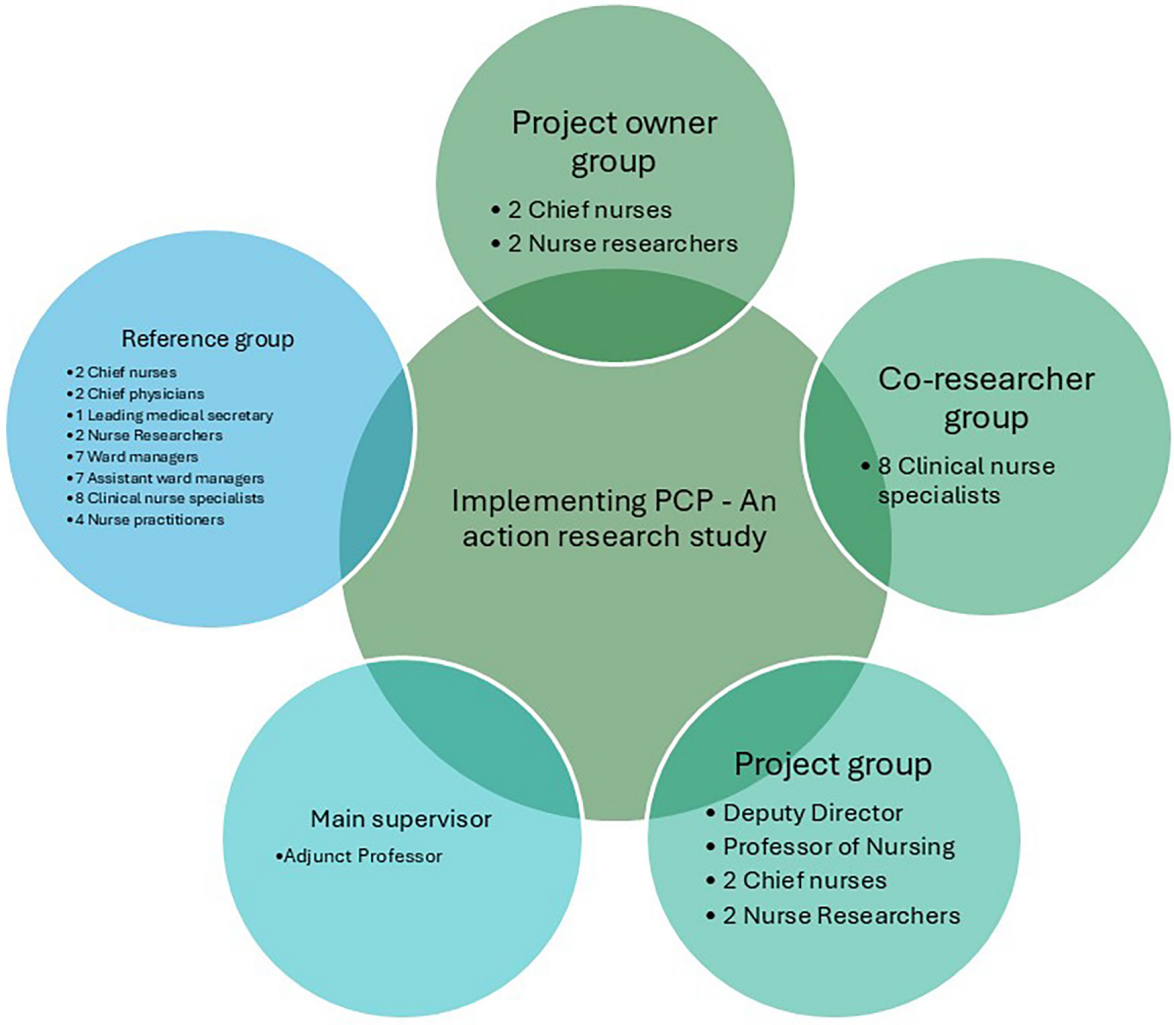
The figure illustrates the organisational diagram of the study.

To uncover perspectives on person-centred practice that may not have otherwise surfaced, individual and collective dialogues and reflections were utilised. The study was governed by a Project Owner Group, a Project Group, a Co-researcher Group, a Reference Group acting as critical friends and a main supervisor. The Project Owner Group was responsible for planning the study and for all final decisions. Additionally, they were responsible for ongoing evaluation of study activities in the cyclic processes and for providing inspiration to the co-researchers and the reference group. The Project Group was responsible for ensuring organisational coherence, sharing knowledge about other relevant projects and addressing interdisciplinary needs for information. The Co-researcher Group served as an important link to clinical practice for the researchers, and they had an important role supporting and facilitating the implementation of PCP by establishing formal as well as informal meetings with discussions on PCP, time for common reflection and dialogues with clinical nurses. Together with the researchers they reflected on evaluation results from the cyclic processes in relation to facilitators and barriers, and in collaboration activities for planning next steps. The Reference Group acted as a critical friend to the Project Owner Group by posing questions, reflecting on evaluation results from the cyclic processes, PCP in clinical practice and providing suggestions for activities and actions aimed at fulfilling the project's purpose and aim. The Reference Group represented collegial perspectives concerning the project. The study organisation diagram is illustrated in Figure 1.

Data collection towards the evaluation of the study outcomes was undertaken with study participants, who were clinical nurse specialists and nurse leaders as they were responsible for translating the PCP framework into clinical practice, as well as clinical nurse practitioners and patients. There were 8 clinical nurse specialists, 7 assistant ward managers, 7 nurse ward managers, 2 chief nurses, 20 clinical nurse practitioners and 30 patients.

### 3.3 Data collection

To capture both the process and outcomes of the study, data were collected through a combination of field studies, interviews and questionnaires. The collected data are detailed in Table 2.

**Table 2:** Flow diagram illustrating the phases, the process methods and data collection used during the study.

Phase 1 (2020–2022)	Phase 2 (2020–2022)	Phase 3 (2022–2023)	Phase 4 (1–2 months of 2024)
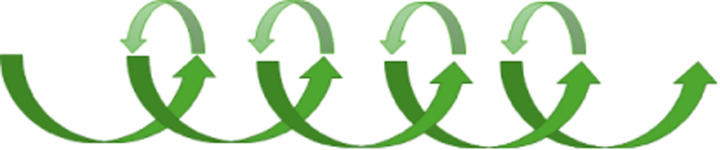			
Baseline-data • **Dialogue meetings** clarifying project expectations with stakeholders (Project owner group, Project group and the Adjunct Professor). ▪ Meeting minutes ▪ Reflective field notes • **Translation and validation of the Person-centred Practice Inventory questionnaires** • **Baseline data** describing patient experiences of care and nurses' level of burnout. ▪ Patient satisfactory surveys (Nationwide survey).	Action-planning • **Workshops on PCP** for chief nurses, ward managers, assistant ward managers, clinical nurse specialists, nurse practitioners and researchers. ▪ Reflective field notes on dynamics during the workshops. • **Field studies** with the purpose of observing the specific clinical practice in relation to PCP and a healthful culture. ▪ Reflective field notes • **Dialogue meetings** with Project owner group, Project group and Adjunct Professor. ▪ Reflective field notes ▪ Meeting minutes	Implementation • **Workshops on PCP** for chief nurses, ward managers, assistant ward managers, clinical nurse specialists, and researchers. ▪ Recordings ▪ Reflective field notes • **Formal and *ad hoc* Dialogue meetings** and conversations with Co-researcher group, Project owner group, Project group and Adjunct Professor. ▪ Reflective field notes ▪ Recordings ▪ Meeting minutes • **Observing workplace cultures** in relation to a healthful culture. ▪ Ad hoc field studies. ▪ “Workplace Culture Critical Analysis Reviser” (WCCAT). ▪ Reflective field notes.	Evaluation • **Evaluating** PCP and PCP Culture as well as the whole project process together with all participants. ▪ Focus group interviews. ▪ Validated questionnaires targeted PCP: Person-centred Practice Inventory—Staff (PCPI-S) and Person-centred Practice Inventory—Care (PCPI-C) questionnaires. ▪ Patient satisfactory surveys (Nationwide survey).

Through the whole process, there was a continuous and dynamic cyclical movement between all the phases.

Text in green illustrates data collection.

In addition to data from the formal meetings we conducted *ad hoc* field studies continuously throughout the whole study period, approximately five hours per week (=45 weeks per year in three years = 650 h).

Furthermore, we wrote reflective notes in relation to the *ad hoc* field studies, which were condensed and transcribed as text in a word-format, equivalent to approximately 75 pages.

Data to explore the experience of engaging with and participating in the project (process data) were collected through field studies during both formal and informal meetings, reflective notes, meeting minutes and recordings of meetings. In clinical practice, we used the Workplace Cultural Critical Analysis Tool (WCCAT) (26). The WCCAT is a participant observation tool developed to capture evidence about workplace culture, it is consistent with the philosophy and values of emancipatory practice development and is a facilitative process (27), see Table 2. All data were collected by the two nurse researchers. All recorded meetings discussions and interviews were transcribed verbatim for analysis.

Data collected in relation to the outcomes achieved from implementing PCP were nurse practitioners' and patients' experiences with the PCP culture. To assess nurse practitioners' and patients' experiences, 20 nurse practitioners and 30 patients from the oncological inpatient ward completed a questionnaire at the end of the study as part of the final evaluation. In phase one, data were collected from the hospital's official patient satisfactory surveys in phase four, data were collected by focus group interviews (Supplementary Appendix S2) and validated questionnaires. Interviews were transcribed verbatim for analysis.

As part of a local study in the Department of Oncology and Palliative Care, questionnaires targeting patients and nurses' experiences of a PCP culture were distributed at the end of the study period. The questionnaires used were the Person-centred Practice Inventory—Staff (PCPI-S) and Person-centred Practice Inventory—Care (PCPI-C) (28, 29). The PCPI-S was developed to measure the experiences of person-centred practice from the perspective of caregivers and items were derived from a consensus-based process with experts on person-centredness described by Slater et al. (29). It consists of 59 items covering all constructs in the five domains of the Person-centred Practice Framework. The PCPI-C measures the experience of person-centred care from the perspective of care receivers/patients (28). The PCPI-C consists of 18 items designed as statements covering the construct of the Person-centred Processes domain of the Person-centred Practice Framework (29). In both questionnaires, each item is presented as a statement and scored on a 5-point-Likert scale ranging from strongly disagree—disagree—neutral—agree—strongly agree. Both instruments have been tested for face validity and are psychometrically valid (28, 29). As nurses' and patients' experiences with nursing care were documented in the Department of Haematology in several local studies there was no need for further questionnaires.

### 3.4 Data analysis

Outcome data from the meetings, field observations and interviews were analysed using thematic analysis in accordance with Braun and Clarke's six phases (30). It is an accessible and theoretically flexible method of qualitative analysis that gives the researcher a method to systematically identify and organise data, in a way that provides insight into themes across the data set. The six phases were (1). Familiarisation with the data, (2). Generating the initial codes, (3). Searching for themes, (4). Reviewing themes, (5). Defining and naming themes and related subthemes, (6). Producing the report. The approach should be viewed as a recursive process (30). The analyses were conducted by the two researchers and the themes were presented to the co-researchers, reference group and the project owner group who were given a chance to review and validate them.

Outcome data from the questionnaires were analysed using descriptive statistics. Data analysis was performed using SPSS, version 21.0 (IBM Corp, Armonk, NY, USA).

Data from the cyclic processes, for example, field observations from clinical practice or recordings from meetings, were primarily analysed by the researchers using meaning condensation (31). Results in relation to the development of PCP were continuously presented at dialog meetings during the process. The results were discussed with co-researchers, reference group and nurse managers in relation to what helped, what hindered and what promoted the development of a PC culture. An example from clinical practice is that through field studies we discovered that the daily operations and *ad hoc* tasks made it difficult for the clinical nurse specialists to take time to facilitate PCP in practice in the care group. As a result of this, the facilitation of PCP in a busy daily practice schedule became a theme at the next joint dialogue meeting, where it was decided that the clinical nurse specialists were given responsibility and influence over the management of their own time.

### 3.5 Ethical considerations

All healthcare professionals in the participating wards were informed about the project. All co-researchers were informed verbally about the purpose of the project and their rights according to participation, as both the hospital management and chief nurses gave their permission for the study to be undertaken, participants were not asked for written consent. As the study did not include sensitive bioinformatic data, and which aims to systematically acquire knowledge about the occurrence or treatment of disease, diagnostics, prevention, rehabilitation of humans, as well as human biological, physiological or psychological processes and heredity it did not require The Danish Ethic Committee's approval. The study was registered with the Danish Data Protection Agency (ref: REG-079-2022).

## 4 Results

The action research study was carried out over a period of four years but interrupted by the COVID pandemic that locked down physical meeting activities from March 2020 to February 2022 when all restrictions were lifted. Results in relation to the study process will firstly be reported and then results in relation to the outcome. Analyses of the concluding interviews with clinical nurse specialists, ward managers, assistant ward managers and chief nurses generated five themes, three in relation to the process and two in relation to the outcome:

1. The PCP framework is complex and difficult to understand and get hold of.
2. When implementing a person-centred framework, the overall context of the departments is important.
3. Psychological safety is important.
4. We have learned a lot in relation to PCP.
5. Values and Beliefs.

### 4.1 Results in relation to the study process

#### 4.1.1 Overview of the process

In total, the Adjunct Professor (overarching project supervisor) visited the departments for ten days. During those days the Inaugural Seminar was held, visits to all wards took place, six workshops and four meetings with ward managers, assistant ward managers, clinical nurse specialists and the researchers took place. A detailed overview of the four project years is illustrated in Supplementary Appendix S1.

During the study, the project owner group held 30 meetings, the project group met eight times, three meetings were replaced by informative e-mails, the reference group was convened for nine meetings of which four were cancelled because of lack of participants, and the co-researchers met ten times. All together more than 80 scheduled meetings and workshops were held.

**Figure 2:**
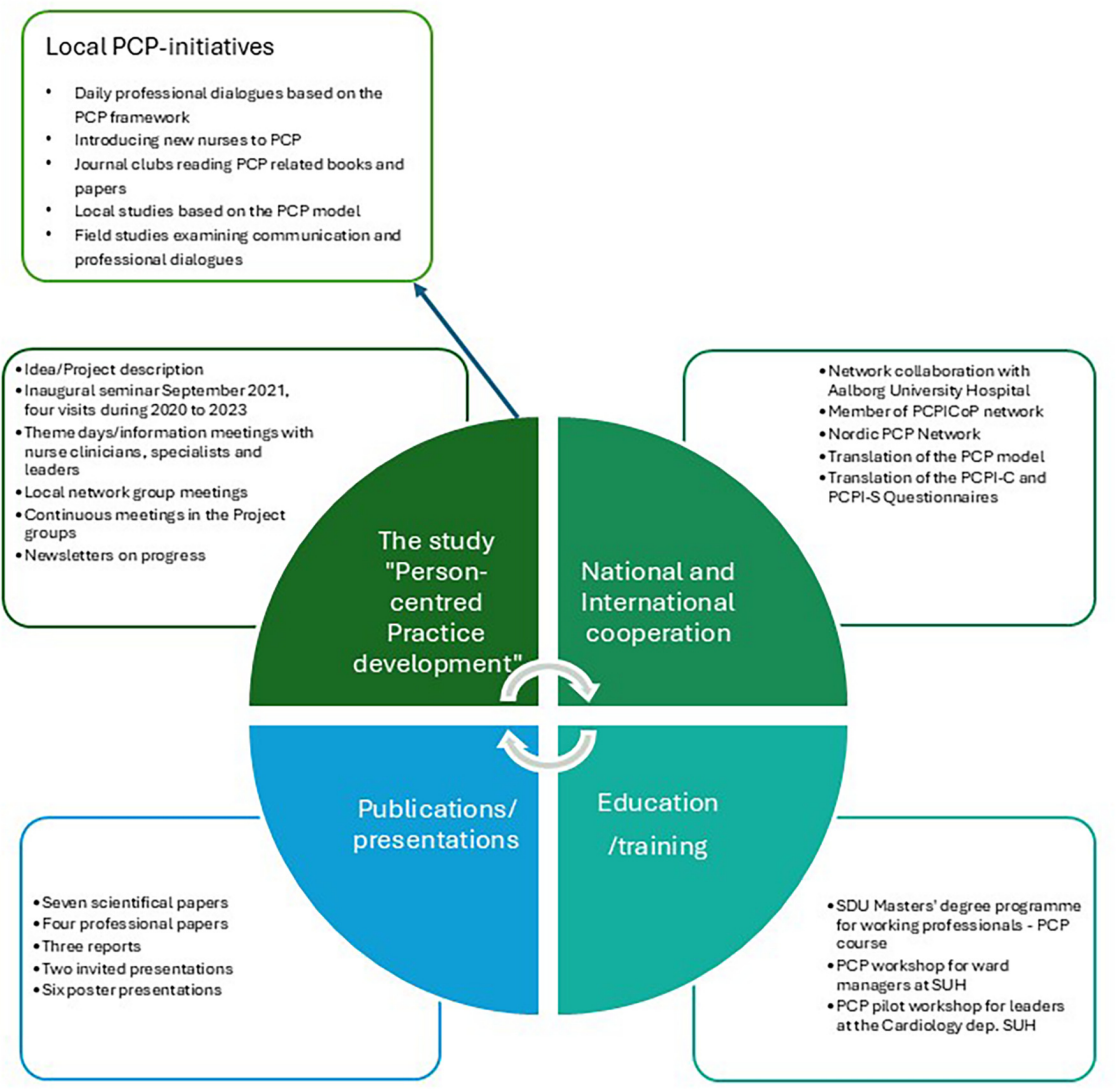
Illustrating the derived outcome from the study “Person-centred practice development” local, national and international.

The study resulted in local initiatives with a PCP approach like training of new employees, reflection meetings, journal clubs, local projects, study units for nursing students, illustrated in Figure 2. Results that went beyond the two departments were the possibility to cooperate with other researchers both national and international. Another derived outcome was establishing continuing education in cooperation with The University of Southern Denmark in creating a master's degree on PCP. A more detailed overview of derived outcome is shown in Figure 2.

#### 4.1.2 Clinical nurse specialists' and leaders' experiences of the implementation process

“The PCP Framework is complex and difficult to understand and get hold of”

Both clinical nurse specialists, ward managers, assistant ward managers and chief nurses agreed that the PCP Framework was complex and understanding it was difficult. This made it hard to implement PCP in clinical practice. Workshops, the development of teaching materials, targeted PCP and facilitation in clinical practice, translation of the PCP Framework, dialogue meetings and newsletters made PCP come alive and the model make more sense. One nurse put it like this

“Only now [after 3 years] PCP has moved in “under my skin”, so that I can now use it and implement it in clinical practice”. (Clinical nurse specialist, Oncological Department)

It was clear from all participants that, it has taken almost three years to get hold of the framework and be able to facilitate PCP processes in clinical practice. All participants agreed that

“It is not until now that I have an understanding of the PCP Framework, that enables me to facilitate PCP processes”. (Clinical nurse specialist, Oncological Department)

“When implementing a person-centred framework, the overall context of the departments is important”

Clinical nurse specialists and ward managers highlighted that there were missing elements of the overall contextual framework especially within the practice environment. For example, time to study and discuss PCP was limited, PCP was not an interdisciplinary initiative as it was introduced solely to nurses, there was a lack of supportive organisational systems as the electronic medical record could not accommodate narrative notes, and democracy was not practiced in teams. All participants recognised that “*The overall context is very important*” (Ward manager, Haematological Department). The clinical nurse specialists, assistant ward managers and ward managers expressed lack of democracy like this

“The decisions are “top down” driven, made by the chiefs and directors and our dialogues are pseudo-democratic—they hear what we have to say but they don’t take it into account” and “they want to control everything”. (Ward manager, Haematological Department)

“Psychological safety is important”

All participants agreed that psychological safety is important, but not all agreed that it was present in the departments. In some situations, participants felt it was difficult to be honest in complicated discussions. A concern about sanctions was expressed and experienced by some. The chief nurses experienced that psychological safety was present between nurses and leaders in the departments—and in addition they stated that “*the tone is important when discussing delicate issues*” (Chief nurse, Haematological Department). To gain psychological safety a safe space for conversations seems crucial. To some participants the safe space was present when they were together with colleagues who have the same role e.g., clinical nurse specialists, ward managers or chief nurses, but not always when leaders were present. Another aspect of psychological safety was the managers concern for the staff. They expressed being aware of not hurting or exposing a staff member in front of other staff members and this resulting in dishonest conversations.

### 4.2 Results in relation to the outcome of the process

#### 4.2.1 Clinical nurse specialists' and leaders' experiences of PCP and the culture after the implementation process

“We have learned a lot in relation to PCP”

Results show that participants gained insight and competences according to the PCP Framework. This resulted in reflection in and on their own practice using the PCP framework. They also became even more aware of the importance of healthful relationships not only to the patients, which they found easy to establish, but also regarding a heightened awareness of inappropriate patterns of behaviour in relation to uni- and interprofessional collegial relationships. This was expressed in

“We have become wiser every day, and one of the things that has developed the most is our attitudes towards other persons. That it is very much about values”. (Ward manager, Haematological Department)

Even though the ability to reflect in and on practice was considered of utmost importance, it was expressed that the ability to reflect differs and varies from person to person.

“Values and beliefs”

All participants expressed the importance of being aware of both their own and shared values and beliefs. The clinical nurse specialists experienced that they did not know what values were important to their colleagues. They had verbalised their own values during the workshops e.g., trust, respect, tolerance, cooperation, fairness, reliability, kindness and trustworthiness but they did not decide on shared values and beliefs. The clinical nurse specialists expressed

“that we may need to be more conscious about our shared values and beliefs”. (Clinical nurse specialist, Oncological department)

Common to clinical nurse specialists, assistant ward managers and ward managers was that they experienced a difference in the espoused values in the departments visions and the values experienced in practice. This was expressed as

“We don’t act person-centred—we know the values for acting person-centred, but our relations and cooperation are not based on respect, fairness and recognition for each other”. (Clinical nurse specialist, Haematological department)

In contrast to this, the chief nurses expressed that the fundamental values and beliefs were experienced as explicit. They stated that

“We already know the values and beliefs we build our nursing upon—We need to focus on the positive narratives”. (Chief nurse, Haematological department)

#### 4.2.2 Nurse practitioners' and patients' experiences of nursing care

**Figure 3:**
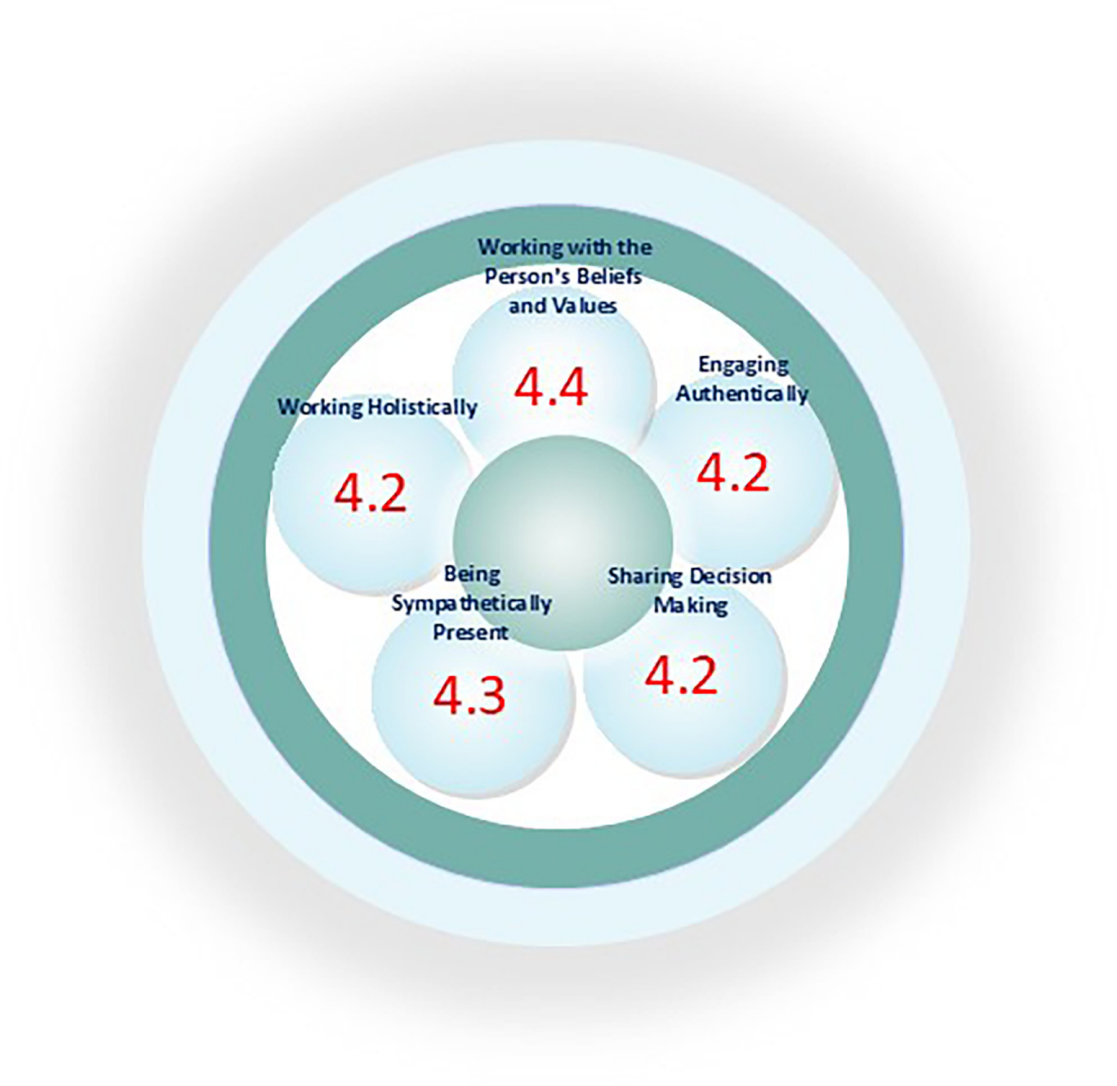
Patients' experiences' of nursing 3½ years after study start.

To assess nurse practitioners' experiences with their care, 20 nurses completed the PCPI-S questionnaire with questions addressing all constructs from the three domains Prerequisites, the Care Environment and the Person-centred Processes of the PCP Framework. Of the 20 nurses who completed the questionnaires 19 were women, two worked for <1 year at the department, seven worked for 1–5 years, and 11 for more than 10 years. Seventeen had a bachelor degree, two a diploma and one had a master degree. Results show that nurses possessed the prequisites for working in a person-centred way, such as being professionally competent, having developed interpersonal skils, being committed to their job, and knowing themselves as persons. Being clear about beliefs and values got the lowest score within the domain with a mean score of 3.8. In relation to the Care Environment, nurses experienced a favourable skill mix, but for the rest of the constructs within this domain the mean score were 3.9 or lower with the lowest mean score of 3.5 to Supportive Organisational Systems. In relation to the Person-centred Processes, the nurse practitioners scored a mean 4.0 to 4.5. Figure 3, illustrates the mean scores of the constructs.

Results from the hospitals general patient satisfactory surveys showed that patients were very satisfied already before the study began with a mean score of 4,3 out of 5. At the end of the study it had raised to a mean of 4.5.

Patients' experiences of the nursing care was assessed using the PCPI-C questionnaire within the five constructs of the Person-centred Processes domain being “Working with Patients Belief and Values”, “Shared Decision-making”, “Engagement”, “Having Sympathetic Presence” and “Providing Holistic Care”. Results show that patients were very satisfied with nursing care as the scores ranged from a mean of 4.2 to 4.4 (standard deviation 0.44–0.59). Results are illustrated in Supplementary Appendix S3.

## 5 Discussion

The findings reveal that the action research processes and methods were meaningful and relevant when developing a PCP culture. All participants gained knowledge and awareness in relation to PCP during the workshops, meetings and reflections with each other, and at the end of the study they experienced being able to facilitate PCP processes in clinical practice. The findings also reveal that nurse practitioners and patients experienced care in accordance with the PCP Framework. By introducing PCP to management, insisting on running the study and affiliating an expert professor, the researchers influenced the context, locally and individually and thus the development of a more PC culture.

The overall results show that the contextual factors have a decisive influence on the development processes and outcomes. The data shows that the participants experienced contradictions between the departments' values and the contextual framework and conditions in relation to exercising PCP.

The two themes we will discuss are (1) clinical nurse specialists' and leaders' experiences of the culture after the implementation process and (2) values and beliefs. We will discuss the two themes under one interconnected theme—*Contextual factors influencing values and beliefs*, especially the experienced discrepancy in espoused values and values experienced in clinical practice.

“Holding the person's values central in decision-making is essential to a person-centred approach in practice (…). Of course, practicing in this way poses challenges to healthcare practitioners who are largely educated and trained in a culture that emphasizes professional control and expertise derived from autonomous decision-making” (23).

Common to clinical nurse specialists, assistant ward managers and ward managers was that they experienced a difference in the espoused values in the departments' vision and PCP framework, and values as they came to light in clinical practice. This was expressed as

“We don’t act person-centred—we know the values for acting person-centred, but our relations and cooperation are not based on respect, fairness and recognition for each other”. (Clinical nurse specialist, Haematological department)

One of the reasons for this clash may be the organisation's values that clash with person-centred values. In another Danish explorative research study focusing on nurses knowledge and behaviour in relation to securing continuity in care trajectories of hospitalized patients (32) a key finding was that even though the nurses had knowledge and wanted to secure continuity, they didn`t succeed due to the hospitals prevailing focus on a biomedical approach to patients, productivity and economics. From an international perspective, McCormack et al. (23) support the findings of this Danish study, with the following quote:

“Being person-centred in a healthcare system that is dominated by business models of efficiency is a challenge for most practitioners. Holding the person at the center of decision-making, when systems increasingly focus on productivity, places person-centredness in a precarious position in the minds of many practitioners” (23).

Another reason for gaps between espoused values and values experienced in clinical practice may have been, that changing a practice culture generates vulnerability and is resource-intensive. It may also be difficult to change one's own behaviour even though there is a desire to (33). McCormack et al. (23) suggests

“Person-centred risk-taking is one of the biggest challenges that practitioners face in working in a person-centred way (…) Working in a person-centred way requires both personal bravery and supported development to make the necessary changes”.

Kristiansen & Bloch-Poulsen suggest that participatory action research might be an offer that employees cannot refuse, given its humanistic and democratic foundation (34). Based on these findings, it can be postulated that even with the existence of a systematic and rigorous study methodology using authentic and targeted evaluation methods, these may be insufficient in ensuring that all participants experience a transformation of clinical practice, or a change in culture. This hypothesis is also supported by other studies (23, 35). Changing a culture is not an easy task and there are no easy ways for this, but awareness, articulation and discussion of values and beliefs and differences in these both at a local and organisational level are an important step in the process. Culture change requires significant and deep change of patterns in organisational systems and approaches that are founded on humanistic principles and values like PCP. It requires an ongoing and sustained commitment to culture enhancement through participatory, collaborative and inclusive development methods (3).

Nevertheless, despite some of the participants experiences whereby they had only little room for PCP, the questionnaires, dialogues and letters from patients showed that they were very satisfied and experienced a high level of PCP. Local patient satisfaction surveys and dialogues with nurse practitioners also showed, that they experienced a healthful culture in the departments. So, despite the participants' experiences of a culture that in several ways and areas did not support a person-centred culture, the experiences from the nursing staff and patients showed another picture. Maybe because the participants level of reflection, due to their involvement in the study and action research processes, had a strong focus on the gaps instead of all that had already been achieved in the development of PCP?

### 5.1 Strengths and limitations

The study represents a cultural development in two cancer departments over a four-year period. To collect data, we used a multi-method approach, illustrated in Table 2, that provided a nuanced picture of all elements of the processes. The validity criteria of action research are a high quality, critical approach, awareness of one's own influence, and fellow researchers' evaluation of data, among other things (36). All aspects have been subject to the researchers' attention and awareness throughout the entire project process, for instance, through dialogue in the project groups, with the participants, and between the researchers. Including two departments represents a strength, as participants from different clinical practices were able to exchange experiences in relation to implementing PCP, and they supported each other at different organisational levels.Additionally, the study had significant managerial support from the departments' chief nurses, and it was grounded in the board of directors by including a Deputy Director in the Project Group. This provided authority to the study, and we were able to arrange a number of theme days and workshops to ensure that nurses at all levels attended at least once to be informed of the PCP Framework.

A study limitation is that the COVID-19 pandemic postponed the start of the study. We were ready with information workshops and theme days, and all participants were eager to participate when Denmark locked down in March 2020, and all meetings were prohibited. We had to wait until September 2021 to resume meetings, and in the beginning of 2022, we held all information workshops and theme days. This gave the study a considerably shorter time to educate participants and implement PCP. This may have affected the clinical nurse specialists' ability to facilitate PCP learning in clinical practice. A limitation was that patients were not included in the action research process but only in the final evaluation. By including patients in the process we might have gained knowledge about important patient perspectives on nursing care. Patients' perspectives were included in the outcome results. The only starting point for the study were the researchers experience of the PC culture and results from the hospital's general patient satisfactory surveys that showed very satisfied patients already before the study started. More knowledge on healthcare professionals and patients experiences in relation to PCP may have helped track the development.

Another limitation may be that the study only concerned PCP in nursing and only included nurses. Changing a culture takes collaboration across interdisciplinary staff members, and with PCP only being acknowledged by the nurses and, in some cases, opposed by other healthcare professionals, this may have affected the conditions for implementation.

## 6 Conclusion

This study underscores the transformative potential of cultivating a caring culture within healthcare settings, shifting towards a more person-centred approach to care. The development of competencies among both nursing staff and leadership has proven essential in fostering an environment conducive to PCP. Notably, the increased awareness and knowledge surrounding PCP have catalysed the initiation of innovative strategies that enhance clinical practice, ultimately benefiting patient care. Moreover, the study has illuminated critical blind spots, including the disconnect between espoused values and experiences in clinical settings, as well as existing workflow inadequacies. The participatory nature of the action research methodology has been instrumental in translating theoretical concepts of person-centred care into practical application. The revelations concerning leadership values and their alignment with practice further emphasize the need for ongoing development. Leaders have expressed a commitment to continue this journey towards enhancing their leadership behaviours in alignment with person-centred principles. Consequently, a trailing research study is warranted to further explore and solidify these developments, ensuring sustained progress in establishing a truly person-centred culture within the organisation. The implications of this study extend beyond immediate clinical practice. The study's findings advocate for a systemic change in healthcare organisations, encouraging a shift from traditional, hierarchical models of care to more collaborative, inclusive frameworks. This shift necessitates a re-evaluation of existing structures and processes, ensuring they are conducive to fostering a culture of care that prioritises the individual needs of patients. It is imperative for organisations to create safe spaces for brave conversations where staff feel empowered to voice their concerns and contribute to the development of person-centred practices. This participatory approach not only enhances the quality of care provided but also fosters a sense of ownership and accountability among staff. Furthermore, the study highlights the need for continuous education and training programs that equip healthcare professionals with the skills and knowledge necessary to embrace person-centredness. These programs should not only focus on the theoretical aspects of PCP but also provide practical tools and strategies for implementation in everyday clinical practice. By fostering a culture of lifelong learning, healthcare organisations can ensure that their staff remain engaged and informed about best practices in person-centred care.

## Data Availability

The datasets presented in this article are not readily available because of logistical restrictions in sharing varied data. Requests to access the datasets should be directed to Elizabeth Rosted, eros@regionsjaelland.dk.
