# Supplementary material for: Enhancing clinical practice through action research: fostering a person-centred culture in healthcare
Source: Front Health Serv. 2025 Jun 6;5:1583478. doi: 10.3389/frhs.2025.1583478 (PMC12179105; doi:10.3389/frhs.2025.1583478)
Supplement: Supplementary file 1 [file Datasheet1.pdf]

Appendix 1

COVID lockdown started 18th March 2020

COVID lockdown ended February 2022

Table 1. Implementing a Person-centred practice and culture. Overview of all arranged activities

	2020		2021		2022		2023	
	**January to June**	**July to December**	**January to June**	**July to December**	**January to June**	**July to December**	**January to June**	**July to December**
**Workshop hosted by Adjunct professor**				**27th September to 1st October**	**28th-29th March**	**21st-23rd September**	**22nd June**	**21st-22nd September 29th November**
**Workshop theme**				Inagural seminar/ Introductory visits to all wards and sections	Clinical nurse specialists (Facilitation)and ward managers (Leadership)	Two-day workshop (Facilitation)	Online meeting with 1. clinical nurse specialists, 2. ward managers 3. chief nurses	Workshop on Psychological safety/ Visit in Dep. of Oncology and meeting with chief nurse and doctor
**Project owner group meetings**	**16th January 3rd March 27th May**	**22nd September**		**30th August 1st, 3rd and 22nd September 2nd December**	**5th January 9th+10th February 1st+7th March 20th April 25th May 13th June**	**5th September 14th October 2nd+30th November**	**4th January 22nd February 29th March 1st May 6th June**	**21st August 30th August 4th September 9th October 27th October 29th November**
**Project owner group meeting themes**	Planning the study and Associated Professor visit	Discussion of action plan		ICN poster and planning Associated Professor visit, follow-up after visit	Planning focus on PCP and nurses in clinical practice/ Planning Associated professor visit/ Evaluating Associated professor visit	Planning Associated Professor meeting/ Evaluating meeting/ Cancelled/ Follow-up	Planning Reference group meeting nr. 4/ Update/ Planning Reference group meeting nr. meeting 5/ Follow-up and ICN	Planning Associated Professor visit/ Planning co-researcher meeting/ Evaluate meetings/ November meeting cancelled
**Project group meetings**	**10th March**			**28th September**	**28th March 20th June**	**6th+21st September 7th December**	**April**	**August 21st September November**

Appendix 1

**Project group meeting themes**	First virtual meeting with Associated Professor			Dinner	Dinner, Info e-mail	Cancelled/dinner/e-mail info	Cancelled	Mid-term status, project end /Dinner/E-mail info
**Co-researcher group**	**25th February**			**8th October**	**28th+29th March 15th June**	**22nd-23rd September**	**16th March 22nd June**	**21st September**
**Co-researcher group activities**	Theme day PCP (ward managers)			Theme day PCP	Workshop hosted by Associated Professor/ Clinical nurse specialists (Facilitation) Ward managers (Leadership)/ ½ theme day for clinical nurse specialists	Two-day workshop (Facilitation)	Meeting with co-researchers/ Online meetings	Workshop (Psychological safety)
**Reference group**					**11th February 20th June**	**28th September November/ December**	**18th January 22nd May**	**18th September 15th November**
**Reference group activities**						Update, Postponed until 18th January 2023	Cancelled/	Cancelled/ Cancelled (replaced by newsletter)
**Co-researchers and clinical practitioners**				**13th+13th+14th October and 2nd+2nd December**	**8th+15th+ 23rd+24th March**			
**Co-researchers and clinical practitioners**				½ PCP theme days	Two- hours PCP Workshop Naestved/Roskilde			
**Network for clinical nurse specialists**								
**Newsletters**				**December**	**April**	**December**		**December**
**Formel mail information**					**March**		**June**	**5th September**

Appendix 1

					E-mail information including project description and two book summaries		e-mail info/Mid-term status report	Meeting minuts and plan going forward
**ICOP meeting**			**17th June**	**28th October and 16th December**	**3rd February 11th May 14th June**	**25th October 19th December**	**8th February 14th June**	**16th November**
**Extras**	**15th June**			**1st October**	**29th March**	**19th-20th September**		**3rd July 21st September**
	Virtual meeting 2½ hours with Associated professor and Project group			Researchers meet with the Associated professor	Researchers meet with the Associated professor (evaluation and translation of questionnaires)	PCP Curriculum Conference in Edinburg		Researchers meeting with Associated Professor in Montreal, Interview with Professor of Nursing and Associated Professor
**Presentations**							**27th January 12th May**	**3rd-5th July 3rd-4th October**
							Interdisciplinary Symposium/ Workshop together with University Hospital Aalborg	Poster ICN Canada, NCNR Island
