# Supplementary material for: Enhancing clinical practice through action research: fostering a person-centred culture in healthcare
Source: Front Health Serv. 2025 Jun 6;5:1583478. doi: 10.3389/frhs.2025.1583478 (PMC12179105; doi:10.3389/frhs.2025.1583478)
Supplement: Supplementary file 2 [file Table1.docx]

Dear Clinical Nurse Specialists, Ward Managers and Chief Nurses

Thank you for agreeing to participate in an evaluation interview as a conclusion to the PCP action research project. We look forward to discussing your experiences, insights, and reflections in relation to the project and its processes.

The interview will take place as a conversation between you, Elizabeth, and Mette. The interview will be recorded.

**Interview Questions**

**Evaluation of the Project:** In relation to the project's goal: the development of the nursing group's competencies towards a person-centred practice/approach, as well as the development of a person-centred departmental culture.

- What experiences and reflections do you have regarding the above? Is there a difference in the competency development among the various nursing groups (leaders, specialist group, clinical staff)?
- Brendan has repeatedly highlighted the challenges in achieving the goal—particularly challenges related to a lack of psychological safety and the existing culture. What are your reflections on this?

**Evaluation of the Project Processes:** In relation to the goal of the action research approach: learning within and beyond practice, as well as change/development in and of practice.

- What are your experiences and reflections regarding this goal?
- What are your thoughts on the process methods used (including dialogue meetings across various areas/levels, workshops, Brendan's visits)?
- What challenges have you encountered in relation to the action research process/the methods employed (e.g., uncovering your own and others' blind spots and the existing culture in practice)?
- Could/should we have done anything differently—and what?

**Suggestions for Future Activities (Follow-up Research Period):** Do you have suggestions for future activities (both collective and/or departmental) regarding further development towards a person-centred culture?

We appreciate your valuable input and look forward to our discussion.

Best regards,
Mette and Elizabeth, Nurse Researchers
