# Supplementary material for: Enhancing clinical practice through action research: fostering a person-centred culture in healthcare
Source: Front Health Serv. 2025 Jun 6;5:1583478. doi: 10.3389/frhs.2025.1583478 (PMC12179105; doi:10.3389/frhs.2025.1583478)

Nurse practitioners' experiences of nursing care at study phase four

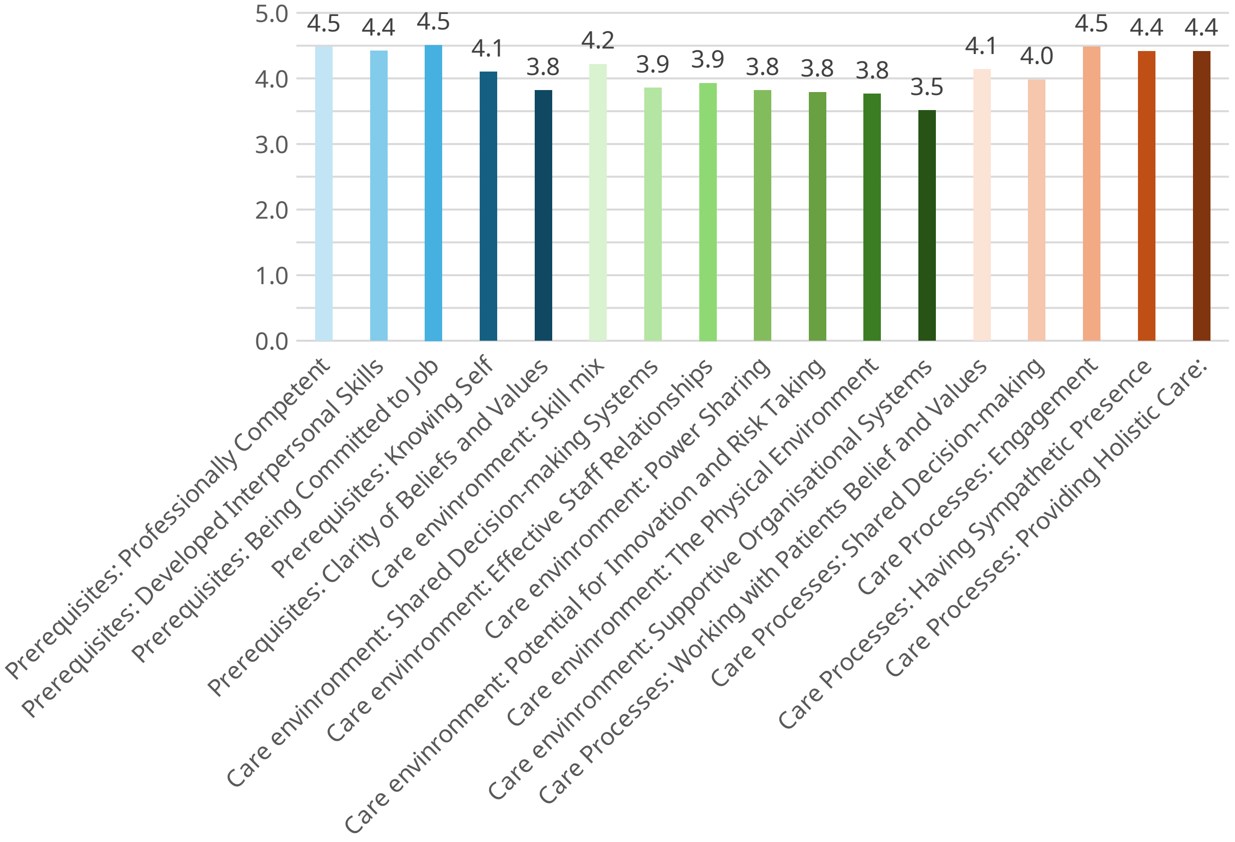

Supplement: Supplementary file 3 [file Table2.docx]
